# Efficient Hole Transfer from CdSe Quantum Dots Enabled by Oxygen-Deficient Polyoxovanadate-Alkoxide Clusters

**DOI:** 10.1021/acs.nanolett.3c02749

**Published:** 2023-11-07

**Authors:** Nicole M. B. Cogan, Kevin P. McClelland, Chari Y. M. Peter, Chayan Carmenate Rodríguez, Alex A. Fertig, Mitesh Amin, William W. Brennessel, Todd D. Krauss, Ellen M. Matson

**Affiliations:** † Department of Chemistry, University of Rochester, Rochester, New York 14627, United States; ‡ Institute of Optics, University of Rochester, Rochester, New York 14627, United States

**Keywords:** colloidal semiconductor nanocrystals, polyoxovanadate clusters, hole transfer, photoluminescence quenching

## Abstract

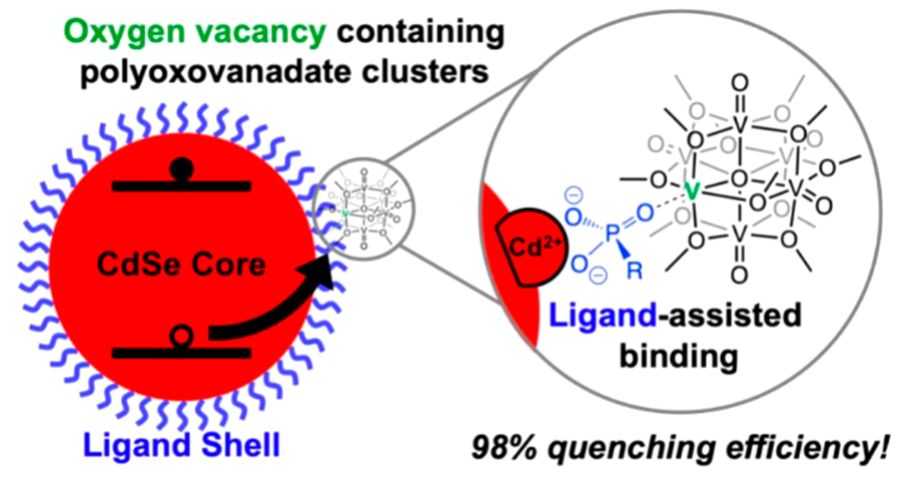

A limitation of the implementation of cadmium chalcogenide quantum dots (QDs) in charge transfer systems is the efficient removal of photogenerated holes. Rapid hole transfer has typically required the *ex situ* functionalization of hole acceptors with groups that can coordinate to the surface of the QD. In addition to being synthetically limiting, this strategy also necessitates a competitive binding equilibrium between the hole acceptor and native, solubilizing ligands on the nanocrystal. Here we show that the incorporation of oxygen vacancies into polyoxovanadate-alkoxide clusters improves hole transfer kinetics by promoting surface interactions between the metal oxide assembly and the QD. Investigating the reactivity of oxygen-deficient clusters with phosphonate-capped QDs reveals reversible complexation of the POV-alkoxide with a phosphonate ligand at the nanocrystal surface. These findings reveal a new method of facilitating QD–hole acceptor association that bypasses the restrictions of exchange interactions.

Cadmium chalcogenide (CdE; E = S, Se, or Te) semiconductor nanocrystals, or quantum dots (QDs), have attracted a great deal of attention as light-harvesting materials for photocatalysis due to their size-dependent bandgaps, high photostability, and large absorption cross sections compared to those of molecular chromophores.^1^ However, the implementation of QDs in photocatalytic systems requires the efficient separation and removal of charges in photogenerated excitons (i.e., dissociation of electron–hole pairs). While the extraction of electrons from the conduction band of photoexcited QDs has been extensively studied, efficient strategies for the transfer of holes remain underdeveloped.^2−4^ Indeed, reductive quenching of cadmium chalcogenide QDs has been identified as a significant barrier in optimizing the efficiency of these materials, given the inherently slow rate of hole transfer and the tendency for trapped holes to etch the nanoparticle surface or decrease colloidal stability through ligand oxidation.^4−6^

Recent work has highlighted strategies for enhancing the rate of hole transfer from CdE QDs.^4,7^ A key design consideration for facilitating charge transfer is association between the QD and the quencher through functionalization of the hole acceptor with a group capable of binding to the surface of the CdE nanocrystal.^8^ However, the binding groups of the quencher are often in competition with coordination of ligands used to solubilize QDs, requiring an additional ligand dissociation step or the addition of large excesses of a quencher. In addition, these functional groups can act as a thermodynamic barrier, inhibiting charge transfer.^9^ To remove this barrier, QD surface capping ligands have been developed that extend the wave function of the nanocrystal outside of the inorganic core and improve the rates of hole transfer to covalently bonded hole acceptors.^10−13^ Others have also shown that efficient hole transfer from CdSe QDs can be achieved through attachment to a hole-accepting scaffold, such as doped V_2_O_5_.^14^ We note, however, that a system that efficiently removes holes from CdE QDs without requiring an intricate organic synthesis or sacrificing the colloidal stability of the QDs is something that remains highly desirable.

Our research team is investigating molecular vanadium oxide assemblies as homogeneous hole acceptors for CdSe quantum dots. These polyoxovanadate-alkoxide (POV-alkoxide) clusters are composed of six vanadyl moieties arranged in a Lindqvist architecture.^15^ Substitution of surface bridging oxides with alkoxide ligands influences the physicochemical properties of the assembly; in addition to solubilizing the vanadium oxide core in an organic solvent,^16−18^ the ligands stabilize vanadium(IV) ions within the Lindqvist ion,^15,19^ rendering electron density available for the reductive quenching of photoexcited CdSe QDs. In previous work, we demonstrated that the addition of [V_6_O_7_(OEt)_12_]^−^ to glutathione-capped CdSe QDs in a 1:1 mixture of ethanol and water enhanced the production of hydrogen from protons.^20^ After determining that the clusters do not function as proton reduction catalysts, we posed the question that catalytic enhancement was a result of an increased level of hole shuttling by the reduced POV-alkoxide. Unfortunately, we were unable to investigate hole transfer dynamics through steady-state or time-resolved photoluminescence (PL) spectroscopies, as [V_6_O_7_(OEt)_12_]^−^ is also capable of accepting electrons from photoexcited CdSe QDs {[V^IV^_5_V^V^O_7_(OEt)_12_]^−^ + e^–^ → [V^IV^_6_O_7_(OEt)_12_]^2–^; *E*_1/2_ = −0.70 V vs NHE in dichloromethane}, complicating analysis.^21^

To exclusively interrogate hole transfer, we needed a form of the POV-alkoxide cluster that would eliminate the possibility of electron transfer from the CdSe QD. Unfortunately, the use of the POV-alkoxide cluster in its fully reduced charge state {e.g., [V^IV^_6_O_7_(OR)_12_]^2–^} was precluded by its poor solubility in nonpolar solvents. Our group has previously reported the synthesis of an oxygen-deficient variant of the Lindqvist ion, [V_6_O_6_(OMe)_12_]^−^ (**V**_**6**_**O**_**6**_^**–**^), that is soluble in dichloromethane.^22,23^ The removal of an oxygen atom from the surface of parent assembly [V_6_O_7_(OMe)_12_]^−^ (**V**_**6**_**O**_**7**_^**–**^) reduces a vanadium(V) center by two electrons, resulting in an oxidation-state distribution of vanadium centers of V^III^V^IV^_5_. Notably, electrochemical characterization of **V**_**6**_**O**_**6**_^**–**^ reveals that the addition of further reducing equivalents to the cluster core is prohibited at energies relevant to electron transfer in CdSe QDs (Figures S1 and S4 and Table S1).^24^

Initial experiments focused on the steady-state analysis of charge transfer between tetradecylphosphonic acid (TDPA)-capped CdSe QDs and **V**_**6**_**O**_**6**_^**–**^ (Figure 1a,c). Remarkably, we found that **V**_**6**_**O**_**6**_^**–**^ quenches the fluorescence of the QDs at a nearly unity efficiency; upon addition of 0.5 equiv of **V**_**6**_**O**_**6**_^**–**^ to a solution of TDPA-capped CdSe QDs in dichloromethane, the PL intensity is decreased by ∼50%, suggesting that nearly every added cluster quenches a corresponding QD. While comparable quenching efficiencies have been observed for statically bound, electron transfer systems,^25^ this amount of quenching is exceptional for a hole transfer system. Even in systems that leverage delocalizing ligands to accelerate hole transfer from the QD core, the addition of 100 equiv of a hole acceptor, resulting in 50 bound equivalents, is necessary to achieve >95% PL quenching.^10^ In contrast, our system can achieve a similar degree of charge transfer with only 10 equiv of **V**_**6**_**O**_**6**_^**–**^, total (Table S2).

**Figure 1:**
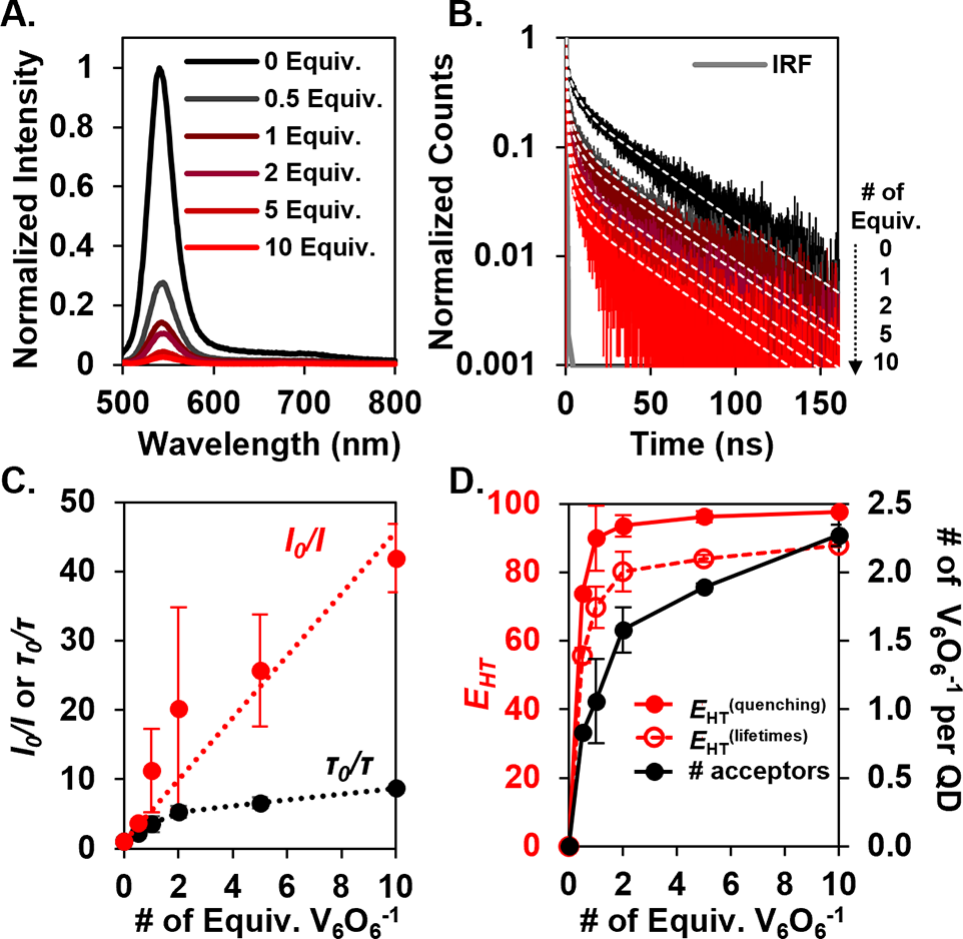
(A) Normalized PL intensity and (B) normalized PL kinetics of CdSe-TDPA QDs with an increasing number of **V_6_O_6_^–^** equivalents. White dotted lines denote the fits using the corresponding parameters listed in Table S2. (C) Concentration-dependent behavior of steady-state PL quenching (red) and changes in PL lifetimes (black). The black dotted line is a guide to the eye, while the red dotted line is a linear fit. τ_0_ is the amplitude-weighted average PL lifetime of just the QDs, and τ is the lifetime with added **V_6_O_6_^–^** (see Table S2). (D) PL quenching efficiency based on steady-state *E*_HT_^(quenching)^ (red) and time-resolved *E*_HT_^(lifetimes)^ (red) measurements and calculated average numbers of acceptors per QD (black, Table S2), which are related to the number of equivalents of **V_6_O_6_^–^**. Error bars are estimated by the standard deviation of multiple measurements. *E*_HT_^(quenching)^ is defined as 1 – *I*/*I*_0_, and *E*_HT_^(lifetimes)^ is defined according to eq 5.^32^

Intrigued by the efficient quenching by **V**_**6**_**O**_**6**_^**–**^, we set out to identify the nature of interactions between the nanocrystal and redox mediator using a combination of steady-state and time-resolved fluorescence quenching studies. The steady-state PL quenching linearly depends on the concentration of **V**_**6**_**O**_**6**_^**–**^, which is typical for such Stern–Volmer analyses, whereby the dotted red line in Figure 1c is a fit to the equation *I*_0_/*I* = 1 + *K*[**V_6_O_6_^–^**], where *K* represents a quenching constant. Using time-correlated single-photon counting techniques (TCSPC) (Figure 1b), we found that with an increasing cluster concentration, the average lifetime of fluorescence for the QDs decreases, suggesting that **V**_**6**_**O**_**6**_^**–**^ participates in dynamic charge transfer with CdSe QDs.^8,26^ Interestingly, we notice that the Stern–Volmer plot obtained from time-resolved PL spectroscopy shows saturation behavior with an increasing number of equivalents of clusters (Figure 1c). This saturation behavior is consistent with a heterogeneous QD–cluster population, which we propose contains weakly associated QD–cluster pairs (but still strongly quenching often termed static quenching), and also free clusters exhibiting weaker dynamic quenching of QD PL.^26^ The weaker dynamic quenching population leads to the expected linear change in PL lifetime with the added cluster, while for the QD–cluster adduct, the nonradiative rate is significantly large such that these QDs are effectively dark. Because dark QDs do not contribute PL photons to the time-resolved PL decay measurement, the τ_0_/τ ratio saturates while the PL quenching linearly increases with added cluster.^27^

The finding that **V**_**6**_**O**_**6**_^**–**^ clusters and QDs are associating is significant, as a key factor facilitating oxidative charge transfer between CdSe QDs and molecular substrates is the ability of the hole acceptor to bind to the surface of the nanocrystal. However, unlike previously reported systems, these clusters do not contain functional groups that would promote the strong binding of the cluster to the surface of the nanocrystal. Indeed, the linear steady-state Stern–Volmer data (Figure 1c) suggest that static quenching arising from a strongly bound QD-cluster adduct is not occurring.^26^

PL quenching could arise from some combination of electron, hole, and energy transfer from the QD to the **V**_**6**_**O**_**6**_^**–**^ cluster. Electron transfer is not expected because the cluster is unable to be reduced by a photoexcited electron on the QD (Figure S1). Nonetheless, we performed ultrafast transient absorption (TA) spectroscopy to probe the excited-state dynamics (see the Supporting Information for experimental details). Note that for core CdSe QDs, with the probe energy set to match the 1S_e_–1S_h3/2_ exciton absorption, TA measurements measure almost exclusively electron dynamics and thus are not sensitive to hole dynamics.^28,29^ Therefore, we expected to see little change in the TA bleach decay dynamics as increasing amounts of the cluster were added. Indeed, the dynamics of the transient bleach over tens of picoseconds for the 1S_e_–1S_h3/2_ exciton is very similar in the presence or absence of cluster (Figure 2). Note that if electron transfer were the operative mode of PL quenching, we would expect to see a significant decay in the TA bleach signal on these time scales, as we have observed for electron transfer to freely diffusing photocatalysts.^30,31^ Energy transfer can also be eliminated as the dominant quenching pathway on the basis of the low degree of spectral overlap between the QD donor and the **V**_**6**_**O**_**6**_^**–**^ acceptor (Figure S2; see the Supporting Information for details of the estimate of a 20% upper bound for energy transfer-related quenching). At longer probe delay times approaching 1 ns, we do see some deviations in the TA signals from pure QDs as 1 and 10 equiv of cluster are added (Figure S3). These differences could be caused by oxidation of the **V**_**6**_**O**_**6**_^**–**^ cluster to **V**_**6**_**O**_**7**_^**–**^ during the TA measurement (thereby allowing for electron transfer to the cluster) and/or some contribution from energy transfer. Importantly, the magnitude of the difference does not nearly account for the amount of quenching that we observe. For example, at 10 equiv of cluster to QD the PL is almost completely quenched [by 90–98% (Table S2)], while the TA data suggest an upper bound on the possible loss of electron population by a factor of ∼2 (Figure S3).

**Figure 2:**
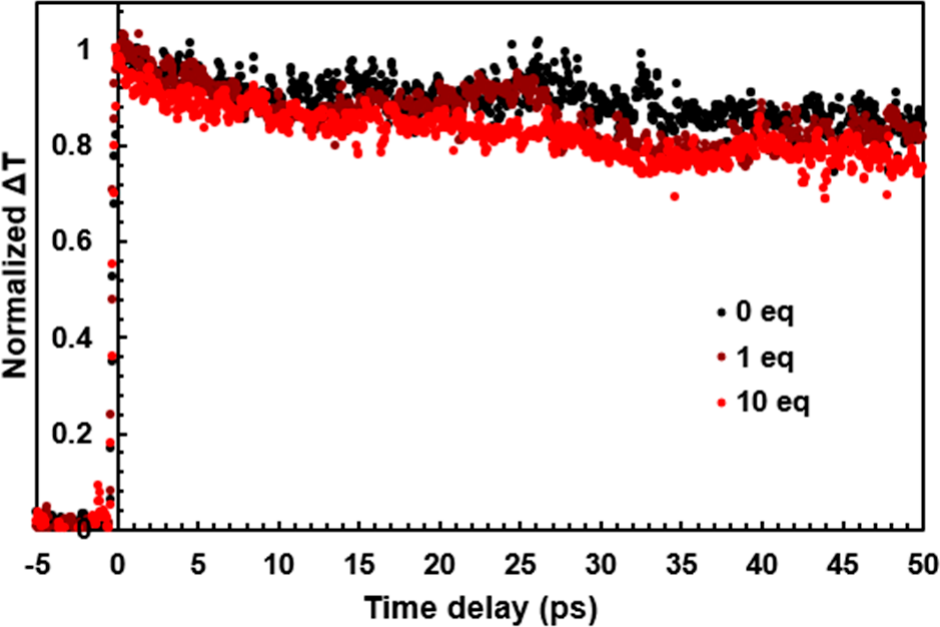
Normalized change in transmission (Δ*T*) of the 1S_e_–1S_h3/2_ transition CdSe QDs without **V_6_O_6_^–^** present (black) and with an increasing concentration of **V_6_O_6_^–^** as a function of probe delay.

To assess the effectiveness of the **V**_**6**_**O**_**6**_^**–**^ clusters to quench QD PL, we modeled the average number of **V**_**6**_**O**_**7**_^**–**^ per QD as described by Tachiya et al. using a Poissonian distribution:^32^


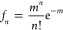
1where *f*_*n*_ is the probability of a given QD being quenched by *n***V**_**6**_**O**_**6**_^**–**^ cluster molecules and *m* = ⟨*n*⟩.^28,30,32−39^ The total rate of hole transfer is simply the product of the effective number of **V**_**6**_**O**_**6**_^**–**^ clusters per QD (*n*) and the hole transfer rate for one cluster per QD:
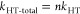
2Altogether, the PL decay of excited QDs at delay time *t* is given by
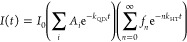
3where *I*_0_ is a normalization constant corresponding to the initial concentration of excited QDs and *A*_*i*_ is the amplitude of the *i*th component of the QD PL decay without a cluster. It can then be shown that the right sum in eq 3 can be simplified resulting in^32^
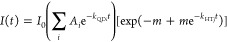
4

The TCSPC PL decay dynamics were fitted using eq 4 to obtain the effective number of **V**_**6**_**O**_**6**_^**–**^ clusters per QD and the HT rate of the 1:1 **V**_**6**_**O**_**6**_^**–**^ to the QD system (see the Supporting Information for a description of the fitting process). The intrinsic QD PL decay is described by a triple-exponential function with the corresponding amplitudes and time constants listed in Table S2. We found that the HT process that arises upon the addition of **V**_**6**_**O**_**6**_^**–**^ to the CdSe QDs system fits to a single exponential with a HT lifetime of 1.4 ns for the 1:1 catalyst:QD system. The amplitudes *B*_*j*_, time constants τ_HT*i*_ (τ_HT*i*_ = 1/*k*_HT*i*_), and average number of cluster acceptors per QD, *m*, for the varying **V**_**6**_**O**_**6**_^**–**^ cluster concentrations are also listed in Table S2. Consistent with the steady-state PL quenching data (Figure 1a), at only 0.5 equiv of **V**_**6**_**O**_**6**_^**–**^, *m* was determined to be 0.83, which suggests that nearly every added cluster is serving to quench PL from a QD (Figure 1d). In addition, while *m* increases monotonically with **V**_**6**_**O**_**6**_^**–**^ concentration, the ability of the added cluster to quench QDs rapidly saturates, plateauing at *m* ∼ 2 for 10 equiv **V**_**6**_**O**_**6**_^**–**^. Additionally, the overall HT efficiency (*E*_HT_) can be estimated by taking the ratio of the integrated area under the time-resolved PL decay curves with and without the cluster according to^40,41^
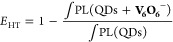
5because the integrated areas of the kinetic decays are proportional to the QD excited electron population. As shown in Figure 1d (and Table S2), the HT efficiency (*E*_HT_) is determined to be 56% for only 0.5 equiv of **V**_**6**_**O**_**6**_^**–**^ and saturates at higher cluster concentrations at *E*_HT_ ∼ 90%. As also shown in Figure 1d, *E*_HT_ is consistent with the PL quenching yield, as determined by the steady-state PL quenching. Note that consistent with Figure 1c, *E*_*HT*_^(quenching)^ > *E*_HT_^(lifetimes)^ with any number of equivalents of **V**_**6**_**O**_**6**_^**–**^.

To identify the mechanism for this QD–cluster association, we next investigated the impact of cluster structure on charge transfer [Figure 3 and Figure S4; all potentials derived from literature or experimentally measured values (Table S1)]. We first compared steady-state PL quenching data of the fully oxygenated assembly, [V_6_O_7_(OMe)_12_]^−^ (**V**_**6**_**O**_**7**_^**–**^), which resulted in a drastic reduction in the quenching efficiency compared to that of **V**_**6**_**O**_**6**_^**–**^ (Figure 3c and Figure S5). The inefficient quenching of CdSe QDs by **V**_**6**_**O**_**7**_^**–**^ strongly suggests that charge transfer to **V**_**6**_**O**_**6**_^**–**^ benefits from some type of coordination mechanism accessed by the defect at the surface of the cluster. To validate the importance of the O-atom vacancy on association with the surface of the QD, we investigated charge transfer dynamics between CdSe QDs and an oxygen-deficient POV-alkoxide cluster functionalized with a 4-*tert*-butylcalix[4]arene (calix) ligand, [^n^Bu_4_N][(calix)V_6_O_6_(OMe)_8_] [**(calix)V**_**6**_**O**_**6**_^**–**^ (Figure 3a and Figure S6)].^42^ This cluster was selected as access to the O-atom vacancy is heavily restricted due to the steric bulk of the calix ligand, prohibiting direct interactions between the surface of the CdSe QD and the defect site. Indeed, fluorescence data in the presence of **(calix)V**_**6**_**O**_**6**_^**–**^ show a quenching efficiency much lower than that with **V**_**6**_**O**_**6**_^**–**^ (Figure 3c), supporting the hypothesis that the strong association observed in the case of **V**_**6**_**O**_**6**_^**–**^ is caused by interaction between the O atom vacancy and the surface of the QD.

**Figure 3:**
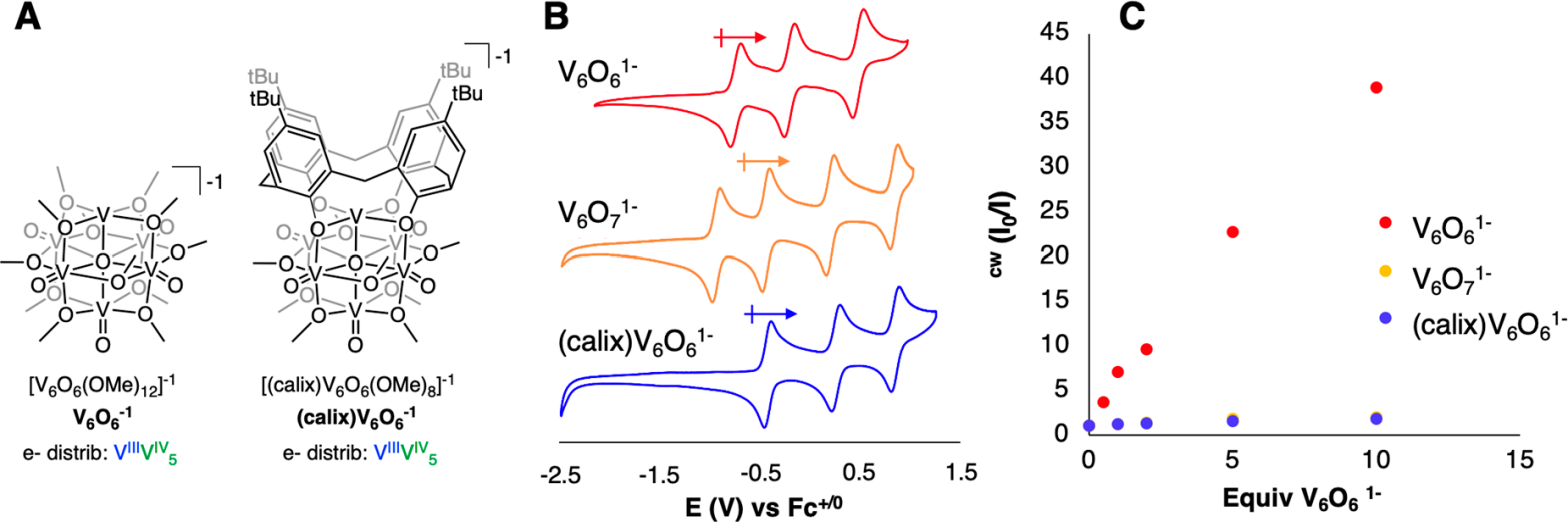
(A) Molecular structures and oxidation-state distributions of the metal ions of **V_6_O_6_^–^** and **(calix)V_6_O_6_^–^**. (B and C) Stern–Volmer plots for **V_6_O_6_^–^** (red), **V_6_O_7_^–^** (orange), and **(calix)V_6_O_6_^–^** (blue) clusters. All potentials are referenced against ferrocene in DCM. All quenching experiments were performed with 1 μM CdSe-TDPA QDs in DCM under inert gas.

Two potential modes of interaction between the TDPA-capped CdSe QDs and the vacancy of **V**_**6**_**O**_**6**_^**–**^ clusters can be envisaged: (i) direct binding of the vacancy to surface Se atoms or (ii) association with TDPA ligands bound to the surface of the QD. Direct coordination to surface selenide moieties was discredited by the lack of reactivity of **V**_**6**_**O**_**6**_^**–**^ with trimethylphosphine selenide (Figure S7); the soft character of selenide renders this Lewis base unreactive with the oxygen-deficient site of the POV-alkoxide. To evaluate the importance of surface ligand identity for cluster adhesion, we synthesized QDs of a similar size that were capped with oleic acid (OA) instead of TDPA. Steady-state PL quenching experiments with OA-capped CdSe and **V**_**6**_**O**_**6**_^**–**^ clusters (Figure S8) show a charge transfer efficiency much weaker than that with TDPA-capped CdSe, indicating that the presence of TDPA is important for the association of **V**_**6**_**O**_**6**_^**–**^ with the surface of the QD.

Given the result that the binding mechanism of **V**_**6**_**O**_**6**_^**–**^ is mediated by the ligand, we hypothesized that the exposed vanadium center interacts directly with the “P=O” moiety of TDPA. This hypothesis was based on our previously reported finding of the coordination of alkyl phosphine oxide compounds to the coordinatively unsaturated vanadium center in oxygen-deficient POV-alkoxides.^43^ To probe the validity of this hypothesis, we next explored the reactivity of **V**_**6**_**O**_**6**_^**–**^ with a phosphonate ester as a surrogate of QD-bound TDPA. Selection of the phosphonate ester eliminates potential reactivity with the acidic protons of the phosphonic acid, which are not expected in a phosphonate ligand bound to the QD surface.^44^ Addition of 2 equiv of dimethoxymethyl phosphine oxide [OPMe(OMe)_2_] to **V**_**6**_**O**_**6**_^**–**^ in dichloromethane results in the instantaneous formation of a new species as observed by ^1^H NMR spectroscopy (Scheme 1 and Figure S9). Unambiguous determination of the molecular structure of **V**_**6**_**O**_**6**_**(OPR**_**3**_**)**^**–**^ was obtained via single-crystal X-ray diffraction; refinement of the data reveals coordination of the phosphate ester to **V**_**6**_**O**_**6**_^**–**^ occurs via binding of the phosphine oxide moiety of the ligand to the oxygen-deficient vanadium center (Figure S10 and Table S3). Notably, electrochemical characterization of **V**_**6**_**O**_**6**_**(OPMe(OMe)**_**2**_**)**^**–**^ shows an electrochemical profile similar to that of **V**_**6**_**O**_**6**_^**–**^; no additional reduction events are accessible following coordination of the phosphine oxide moiety (Figure S1), indicating that hole transfer remains the sole charge transfer mechanism available upon complexation of the cluster to the TDPA ligands of the QD.

**Scheme 1:**
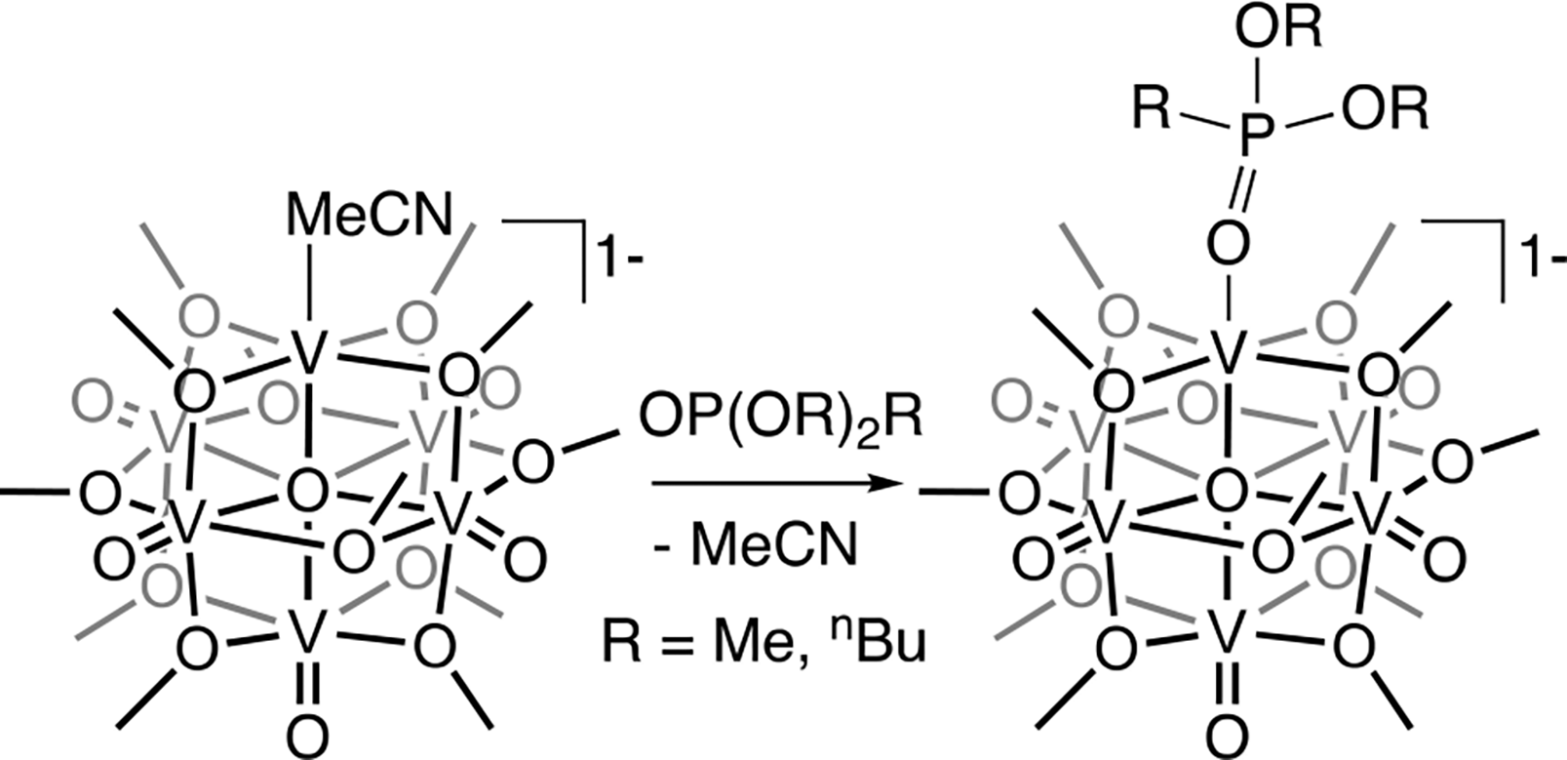
Synthesis of **V**_**6**_**O**_**6**_**(OPR**_**3**_**)**^**–**^

Quantitative conversion of **V**_**6**_**O**_**6**_^**–**^ into **V**_**6**_**O**_**6**_**(OPMe(OMe)**_**2**_**)**^**–**^ following the addition of only 2 equiv of phosphate ester in dichloromethane is significant for understanding the near unity quenching of CdSe QDs by the oxygen-deficient POV-alkoxide. As this number is significantly lower than the expected number of phosphonate ligands required to keep the QDs colloidally stable (∼10^2^),^45^ we can infer a strong association of **V**_**6**_**O**_**6**_^**–**^ with TDPA-capped QDs at concentrations studied in this report. We hypothesize that binding of the ligand to the CdSe-QD remains unchanged upon introduction of the cluster; the bidentate nature of the phosphonate ligands leaves P=O functional groups accessible to complexation with **V**_**6**_**O**_**6**_^**–**^. Quantitative formation of a phosphonate ester adduct of the oxygen-deficient POV-alkoxide cluster is also observed when using a bulkier phosphonate ester [e.g., OP^n^Bu(O^n^Bu)_2_ (Figure S11)], suggesting that the sterics of the components bound to the phosphonate ligand do not have a substantial impact on the affinity of the phosphine oxide moiety of **V**_**6**_**O**_**6**_^**–**^.

The ligand-based association mechanism reported here is interesting, as most binding interactions with the surface of a QD require the *ex situ* functionalization of a binding group to a quencher followed by a competitive exchange interaction between the substrate and ligands.^10^ As phosphonate ligands form a very stable interaction with the surface of the QD, CdSe-TDPA nanocrystals would normally require an even stronger-binding, thiolate-functionalized quencher to displace the native ligands.^46^ However, thiolates are not appropriate for hole transfer systems as they participate in competitive, irreversible oxidation reactions.^4^*Ex situ* functionalization therefore requires either treatment of the CdSe-TDPA QDs to remove ligands or addition of excess equivalents of the preassociated TDPA-**V**_**6**_**O**_**6**_^**–**^ complex to facilitate cluster association.^47^ The novel *in situ* binding mechanism presented here circumvents these issues by association with already bound ligands rather than their replacement, resulting in a nearly unity quenching efficiency.

## Conclusion

We have shown that oxygen-deficient POV-alkoxides are effective redox mediators for the removal of photogenerated holes from CdSe QDs. We confirmed this through steady-state and time-resolved PL quenching studies of fully reduced clusters where hole transfer is the only available quenching pathway. Introduction of an oxygen atom vacancy drastically improves hole transfer from CdSe QDs to nearly unity quenching efficiency. Interestingly, we conclude that rather than a direct mechanism of binding to surface atoms, these clusters can form a complex with the phosphonate capping ligands on the QDs. This ligand-based binding method of surface adhesion circumvents the limitations of classical competitive exchange mechanisms, resulting in high degrees of quenching at stochiometric cluster concentrations. Our findings, therefore, point to a novel association mechanism between QDs and POV-alkoxide redox mediators that has drastic implications for oxidative chemistry.

## Supporting Information Available

Experimental procedures and details of curve fitting calculations, supporting CV, NMR, and optical spectroscopic data (PDF)
Structural data for **V**_**6**_**O**_**6**_**(OPMe(OMe)**_**2**_**)^−^** (CIF)

## Supplementary Material

nl3c02749_si_001.pdf

nl3c02749_si_002.cif
