# Supplementary material for: Efficient Hole Transfer from CdSe Quantum Dots Enabled by Oxygen-Deficient Polyoxovanadate-Alkoxide Clusters
Source: Nano Lett. 2023 Nov 7;23(22):10221–7. doi: 10.1021/acs.nanolett.3c02749 (PMC10683070; doi:10.1021/acs.nanolett.3c02749)
Supplement: Supplementary file 1 — nl3c02749_si_001.pdf [file nl3c02749_si_001.pdf]

Supplementary Information

Efficient Hole Transfer from CdSe Quantum Dots Enabled by Oxygen-deficient Polyoxovanadate-alkoxide Clusters

Nicole M. B. Cogan[†1], Kevin P. McClelland[†1], Chari Y. M. Peter[†1], Chayan Carmenate Rodríguez[1], Alex A. Fertig[1], Mitesh Amin[1], William W. Brennessel[1], Todd D. Krauss*[1,2], and Ellen M. Matson*[1]

[1] Department of Chemistry, University of Rochester, Rochester, NY 14627, USA

[2] Institute of Optics, University of Rochester, Rochester, New York 14627, USA

[†] Authors contributed equally to this work.

Table of Contents:

Experimental procedures and calculations

Synthesis of TDPA- and OA-capped CdSe QDs S2
Synthesis of POV-alkoxide clusters S3
Energy Transfer Calculations S6
Analysis of PL Decay Dynamics S6

Supporting Figures

Figure S1. Cyclic voltammograms of various POV-alkoxide clusters S4
Figure S2. Absorbance spectra of CdSe-TDPA with $V_6O_6^{-1}$ clusters S6
Figure S3. Transient absorption dynamics S8
Figure S4. Relative potentials of Cd-TDPA and POV-alkoxide clusters S8
Figure S5. Absorbance and photoluminesce spectra of CdSe-TDPA with $V_6O_7^{-1}$ S8
Figure S6. Absorbance and photoluminesce spectra of CdSe-TDPA with (calix)$V_6O_6^{-1}$ S9
Figure S7. Reactivity of $V_6O_6^{-1}$ clusters with trimethyl phosphine selenide S10
Figure S8. Quenching of CdSe-OA with $V_6O_6^{-1}$ clusters S11
Figure S9. ^{1}H NMR spectrum of $V_6O_6(OPMe(OMe)_2)^{-1}$ S12
Figure S10. Crystal structure of $V_6O_6(OPMe(OMe)_2)^{-1}$ S12
Figure S11. ^{1}H NMR spectrum of $V_6O_6(OPBu(OBu)_2)^{-1}$ S14
Figure S12. Reactivity of (calix)$V_6O_6^{-1}$ clusters with phosphonate esters S15

Supporting Tables

Table S1. Summary of redox events for POV-alkoxide clusters S5
Table S2. Summary of fits for TCSPC of CdSe-TDPA with $V_6O_6^{-1}$ clusters S7
Table S3. Table for crystal structure of $V_6O_6(OPMe(OMe)_2)^{-1}$ S13

Experimental Methods

Chemicals: Chemicals were purchased and used without further purification except where noted. Cadmium (II) Acetate anhydrous ($Cd(Ac)_2$, 99.9995%), Selenium (Se, 100 mesh, 99.99%), Trioctylphosphine (TOP, 97%), Trioctylphosphine Oxide (TOPO, 99%), Tetradecylphosphonic Acid (TDPA, 98%), Hexadecylamine (HDA, 98%), Cadmium Oxide (CdO, 99.99%), 1-Octadecene (ODE, 90%), Oleic Acid (OA, 90%), Tetrabutylammonium Borohydride (TBA BH_4, 98%), Vanadium(V) Oxytriisopropoxide ($VO(OiPr)_3$), and Trimethylphosphine (TMP, 97%) were purchased from Sigma-Aldrich. Tetrabutylammonium Hexafluorophosphate (TBA PF_6, 98%) was purchased from Sigma-Aldrich, recrystallized thrice using hot methanol, and stored under dynamic vacuum for a minimum of two days prior to use.

Synthesis of Tetradecylphosphonic Acid (TDPA)-Capped CdSe Quantum Dots (QDs): Inside a nitrogen filled glovebox, 0.79 g of Se was dissolved in 10 mL of 97% TOP in a scintillation vial and allowed to stir at 50 °C until all solid had dissolved. In a separate vial, 0.747 g of anhydrous $Cd(Ac)_2$ was dissolved in 12 ml of 97% TOP and stirred at 50 °C until all solid had dissolved. Outside of the glovebox, to a 100 mL three neck flask 7.8 g of TOPO, 2.3 g of 98% HDA, and 0.171 g of 97% TDPA was added. The flask was purged with N_2 three times, heated to 100 °C, and vacuum was pulled. The flask was left below 0.1 Torr for 30 minutes with stirring. After returning to N_2, 1.8 mL of the TOP/Se solution was injected, and the reaction was heated to 310 °C, where 2.5 mL of the TOP/$Cd(Ac)_2$ solution was injected very quickly with a syringe. The heating mantle was set to 260 °C, and the reaction was allowed to stir for 7 minutes. The heating mantle was then removed and the solution was cooled using a heat gun. At ~100 °C, 15 mL of hexanes was injected into the reaction to prevent solidification of TOPO. The mixture was separated into 2 x 50-mL centrifuge tubes, where 35 ml of methanol was added. The product was centrifuged for 15 minutes at 8000 RPM. The clear supernatant was discarded, and the solid pellet was allowed to air dry. The solid was dispersed in 10 mL hexanes and placed in the refrigerator at 8 °C overnight to precipitate TOPO. The solution was centrifuged for 15 minutes at 8000 RPM. The colored supernatant was transferred to new falcon tubes and the white/pink precipitate was discarded. 45 mL of ethanol was then added to the QD solution and the mixture was centrifuged for 15 minutes at 8000 RPM. The clear supernatant was discarded, and the solid pellet was allowed to air dry. The solid was dispersed in 15 mL dichloromethane. The solution was then degassed via the freeze-pump-thaw method and stored in a glovebox.

Synthesis of Oleic Acid (OA)-Capped CdSe Quantum Dots (QDs): To a dry, three-neck round-bottom flask, 3 mL of OA, 10 mL of ODE and 0.514 g of CdO (0.514 g, 4.00 mmol) were added. The flask was placed under an N_2 atmosphere and heated to 270 °C with vigorous stirring. While the solution was heating, the Se precursor was prepared by sonicating 0.68 g of Se in 10 mL ODE until a uniform suspension was formed. Once the Cd precursor became clear, the temperature was set to 260 °C and 1 mL of the Se precursor was rapidly injected. The QDs were allowed to grow for 60 seconds before thermal quenching with a water bath. In order to remove the unused precursor, the solution was washed with ethanol in a 6:1 (v/v) ratio to QDs followed by centrifugation at 7500 rpm for 10 min. The clear supernatant was then discarded and the pellet was

redispersed in minimal hexanes. We repeated this washing once more and stored the final QD solution in 15 mL dichloromethane.

Synthesis of [^{n}Bu$_4$N][$V_6O_7(OCH_3)_{12}$]: $V_6O_7^{-1}$ was prepared according to previous literature.[1]

Synthesis of [^{n}Bu$_4$N][$V_6O_6(OCH_3)_{12}$]: $V_6O_6^{-1}$ was prepared according to previous literature.[2]

Synthesis of [^{n}Bu$_4$N][$V_6O_6(OCH_3)_{12}OP(OCH_3)_2CH_3$]: A 20 mL scintillation vial was charged with [^{n}Bu$_4$N][$V_6O_6(OCH_3)_{12}$] (**$V_6O_6^{1-}$**) (0.046 g, 0.046 mmol) and 5 mL of DCM. Dimethyl methylphosphonate (10 µL, 0.091 mmol) was added with stirring. A gradual color change from dark red to brown was observed over the course of 10 minutes; the solution was left to stir at room temperature for 1 hour to ensure completion of the reaction. Volatiles were removed under reduced pressure, resulting in the formation of a brown solid. The product was washed with 10 mL diethyl ether to remove unreacted dimethyl methylphosphonate, affording the isolation of the product, [^{n}Bu$_4$][$V_6O_6(OCH_3)_{12}OP(OCH_3)_2CH_3$] (0.053 g, 0.046 mmol, 100%). ^{1}H NMR (400 MHz, CD_2Cl_2): *d* = -15.24, 1.03, 1.41, 3.11, 4.59, 7.05, 24.89, 25.33, 26.69. Elemental analysis: Calcd for $V_6O_{21}C_{31}H_{81}PN$ (MW = 1140.59 g/mol): C, 32.64; H, 7.16; N, 1.23. Found: C, 32.589; H, 6.995; H, 1.106.

Synthesis of [^{n}Bu$_4$N][$V_6O_6(OCH_3)_{12}OP(OC_4H_9)_2C_4H_9$]: A 20 mL scintillation vial was charged with [^{n}Bu$_4$N][$V_6O_6(OCH_3)_{12}$] (**$V_6O_6^{1-}$**) (0.046 g, 0.046 mmol) and 5 mL of DCM. Dibutyl butylphosphonate (26 µL, 0.091 mmol) was added with stirring. A gradual color change from dark red to brown was observed over the course of 10 minutes; the solution was left to stir at room temperature for 1 hour to ensure completion of the reaction. Volatiles were removed under reduced pressure, resulting in the formation of a brown solid. The product was washed with 10 mL diethyl ether to remove unreacted dibutyl butylphosphonate, affording the isolation of the product, [^{n}Bu$_4$N][$V_6O_6(OCH_3)_{12}OP(OC_4H_9)_2C_4H_9$] (0.048 g, 0.038 mmol, 83%). ^{1}H NMR (400 MHz, CD_2Cl_2): *d* = -15.29, 0.91, 1.25, 2.04, 2.34, 2.96, 4.39, 7.08, 25.18, 26.52.

Photoluminescence Quenching: QD samples were placed in a quartz cuvette with a 1 cm path length for all absorption and emission characterization. A PerkinElmer Lambda 950 UV/Vis/NIR spectrophotometer was used to record all absorbance spectra. Following all syntheses and ligand exchanges, the CdSe QD concentration was calculated using the first excitonic absorbance transition as described by Yu, et. al.[3] Photoluminescence (PL) spectra were measured with a modular fluorometer system (Acton Research) with a photomultiplier tube detector.

Cyclic Voltammetry: All experiments were recorded with a Bio-Logic SP-150 potentiostat/galvanostat and the EC-Lab software suite. All measurements were performed in a three-electrode system cell configuration that consisted of a glassy-carbon (ø = 3.0 mm) as working electrode (CH Instruments, USA), a Pt wire as the counter electrode (CH Instruments, USA), and an Ag/Ag^+ non-aqueous reference electrode with 0.01 M $AgNO_3$ in 0.05 M [^{n}Bu$_4$N][PF_6] in acetonitrile (BASi, USA). All electrochemical measurements were performed at room temperature in a nitrogen-filled drybox.

Time-Correlated Single Photon Counting: Ensemble TCSPC measurements were acquired using a home-built optical setup featuring an inverted microscope (Nikon, TE-200U) with a 10x

air objective (Nikon, NA 0.3) and a 488 nm dichroic mirror (Semrock, Inc.). Samples, which had been transferred to quartz cuvettes with 1 mm thick windows, were photoexcited by a defocused laser beam provided by a pulsed laser diode (PicoQuant, PDL-800D) emitting 485 nm light with a repetition frequency of 2.5 MHz. The laser power was adjusted to ensure the photon flux was no higher than 10^{12} photons per cm^2 per pulse. Photoluminescence (PL) emission was collected by a single photon avalanche diode detector (Micro Photon Devices, PDM Series, 50 μm active sensing area diameter) with a fast-timing resolution lower than 100 ps. All components of the TCSPC setup were incorporated in the PicoHarp 300 module (PicoQuant). The instrument response function was collected from residual scattering of the light from the pulsed laser diode. To collect lifetime decay curves, 488 nm and 532 nm longpass filters were placed in the emission pathway to block scattered excitation light. Decay curves corresponding to CdSe-TDPA QDs without the quencher were first fit to a triple exponential decay to establish the dynamics before PL quenching was initiated. Subsequent decay curves corresponding to CdSe-TDPA QDs with the quencher were fit according to the lifetime parameters calculated for the pre-quenching sample and an additional, floating parameter used to examine the quenching process.

Transient Absorption: TA measurements were performed using an ultrafast laser system in a pump-probe configuration. Femtosecond pulses (277 fs) from a 1035 nm diode-pumped fiber laser (Coherent, Monaco IR) at 500 kHz were introduced into an optical parametric amplifier (Coherent, Opera-NOPA). The resulting signal pulses (≈ 50 fs, 500 kHz) were tuned to the desired wavelength (525 nm) for the pump, probe, and reference beams. The reference beam was split from the probe before the sample and used to correct the probe for pulse-to-pulse fluctuations. A motorized delay stage was used to control the optical time delay between the pump and probe pulses. Samples were placed in a 2 mm path length quartz cuvette and stirred to avoid laser-induced signal artifacts. The optical density of CdSe QDs was 0.2 for at the pump wavelength of 525 nm. The average number of excitons absorbed per QD, $\langle N_0 \rangle$, was kept below one for all measurements.

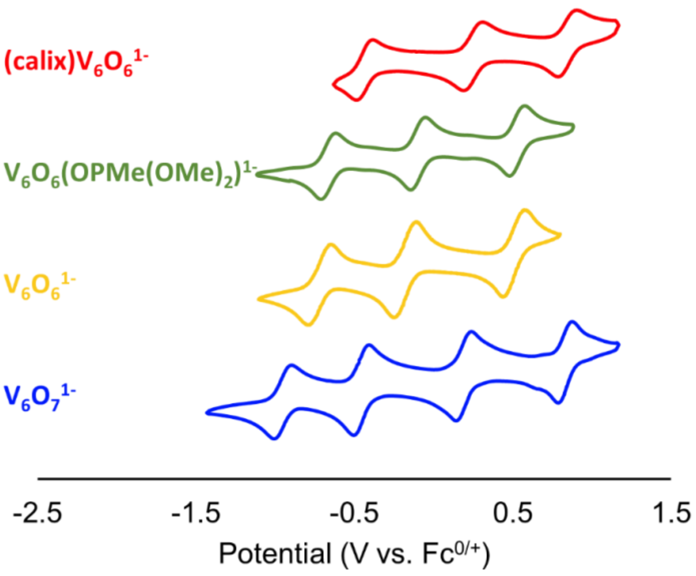

Figure S1. Cyclic voltammograms of **(calix)$V_6O_6^{1-}$** (top), **$V_6O_6(OPMe(OMe)_2)^{1-}$** (second from top), **$V_6O_6^{1-}$** (second from bottom), and **$V_6O_7^{1-}$** (bottom). All samples were run with 1 mM cluster in acetonitrile, with 0.1 M [^{n}Bu$_4$N][PF$_6$] as supporting electrolyte. All potentials are referenced against Ferrocene.

Table S1. Redox potentials for **(calix)$V_6O_6^{1-}$**, **$V_6O_6(OPMe(OMe)_2)^{1-}$**, **$V_6O_6^{1-}$**, and **$V_6O_7^{1-}$** referenced against $Fc^{+/0}$, $Ag^{+/0}$, and NHE. Converting the potentials from being referenced to $Fc^{+/0}$ to $Ag^{+/0}$ was done by $E_{1/2}(\text{vs } Ag^{+/0}) = E_{1/2}(\text{vs } Fc^{+/0}) + 0.65$.[4] Converting the potentials from being referenced to $Fc^{+/0}$ to NHE was done by $E_{1/2}(\text{vs NHE}) = E_{1/2}(\text{vs } Fc^{+/0}) + 0.66$.[5]

**(calix)V_6O_6**	$V^{IV}_5V^{III}/V^VV^{IV}_4V^{III}$	$V^VV^{IV}_4V^{III}/V^V_2V^{IV}_3V^{III}$	$V^V_2V^{IV}_3V^{III}/V^V_3V^{IV}_2V^{III}$	
Vs $Fc^{+/0}$	-0.43698	0.245438	0.841355	
Vs $Ag^{+/0}$	0.213022	0.895438	1.491355	
Vs NHE	0.223022	0.905438	1.501355	
**V_6O_6-OPMe(OMe)$_2$**	$V^{IV}_5V^{III}/V^VV^{IV}_4V^{III}$	$V^VV^{IV}_4V^{III}/V^V_2V^{IV}_3V^{III}$	$V^V_2V^{IV}_3V^{III}/V^V_3V^{IV}_2V^{III}$	
Vs $Fc^{+/0}$	-0.66367	-0.09672	0.528912	
Vs $Ag^{+/0}$	-0.01367	0.553278	1.178912	
Vs NHE	-0.00367	0.563278	1.188912	
**V_6O_6**	$V^{IV}_5V^{III}/V^VV^{IV}_4V^{III}$	$V^VV^{IV}_4V^{III}/V^V_2V^{IV}_3V^{III}$	$V^V_2V^{IV}_3V^{III}/V^V_3V^{IV}_2V^{III}$	
Vs $Fc^{+/0}$	-0.71814	-0.18194	0.504093	
Vs $Ag^{+/0}$	-0.06814	0.468059	1.154093	
Vs NHE	-0.05814	0.478059	1.164093	
**V_6O_7**	$V^{IV}_6/V^{IV}_5V^V$	$V^{IV}_5V^V/V^{IV}_4V^V_2$	$V^{IV}_4V^V_2/V^{IV}_3V^V_3$	$V^{IV}_3V^V_3/V^{IV}_2V^V_4$
Vs $Fc^{+/0}$	-0.95255	-0.4568	0.187021	0.83097
Vs $Ag^{+/0}$	-0.30255	0.193196	0.837021	1.48097
Vs NHE	-0.29255	0.203196	0.847021	1.49097

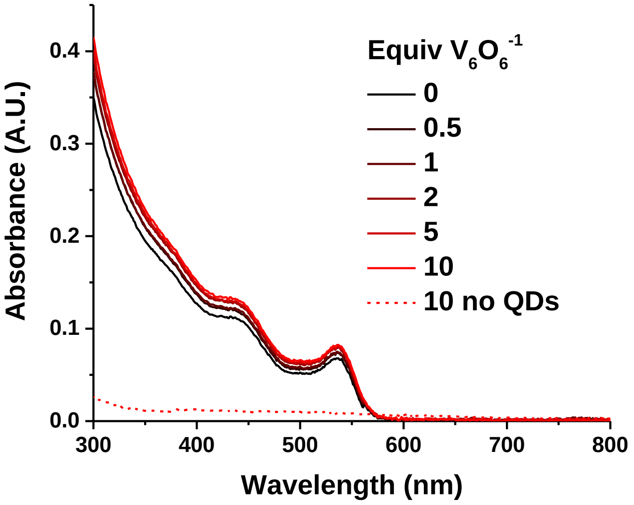

Figure S2. The absorbance spectra of CdSe-TDPA with increasing concentration of $\mathbf{V_6O_6^{-1}}$ (solid black to red) as well as 10 μM $\mathbf{V_6O_6^{-1}}$ without QDs (dashed red). All QD samples contained 1 μM CdSe-TDPA QDs with 0-10 equivalents of $\mathbf{V_6O_6^{-1}}$ in DCM and under inert gas.

Energy Transfer Calculations: As mentioned in the main text, in addition to charge transfer, energy transfer is another potential pathway for fluorescence quenching of the QDs. In order to determine if energy transfer is occurring in this system, we must compare the overlap between the emission of the QDs and absorption of $\mathbf{V_6O_6^{-1}}$ (**Figure S2**) The expected yield of energy transfer, E, can be determined as:

$$E = \frac{1}{1+\left(\frac{r}{R_0}\right)^6} \tag{S1}$$

where r is the center-to-center distance of the QDs and clusters and R_0 is the Förster distance, the distance at which energy transfer efficiency is 50% which can be estimated by:

$$R_0{}^6 = \frac{2.07*\kappa^2*\phi_{QD}*J}{128\pi^5*N_A*n^4} \tag{S2}$$

where κ^2 is the dipole orientation factor (assumed to be 2/3 for these quasi-spherical structures), ϕ_{QD} is the fluorescence QY for the QD without cluster present (0.1, measured previously)[6], N_A is Avagadro's number, n is the refractive index of DCM, and J is the spectral overlap integral in units of M^{-1} cm^{-1} nm^4, calculated as:

$$J = \int \overline{F_D}(\lambda)\varepsilon_A(\lambda)\lambda^4 d\lambda \tag{S3}$$

where $\overline{F_D}(\lambda)$ is the fluorescence of the donor with area normalized to 1, $\varepsilon_A(\lambda)$ is the extinction coefficient[7] of $\mathbf{V_6O_6^{-1}}$ at wavelength λ. From this, we obtain an overlap of 4.62*10^13 M^{-1} cm^{-1} nm^4 and a resulting R_0 of 2.01 nm. This value is quite small given the minimal cluster absorbance (**Figure S2**). Assuming the smallest center-to-center distance possible of 2.5 nm,[3, 8] we achieve a

“best case” E of 21% which does not account for the ~ 50% quenching observed with 1 equivalent added (**Figure 1**), indicating that the majority of quenching is due to charge transfer.

Analysis of PL Decay Dynamics: As described in previous reports,[9] the intrinsic QD dynamics were obtained by fitting the kinetics of QDs without clusters to yield the amplitudes A_i and time constants τ_{QDi} ($\tau_{QDi} = 1/k_{QDi}$). The kinetics of the QDs with various $\mathbf{V_6O_6^{-1}}$ cluster concentrations was globally fit, holding the amplitudes A_i and rate constants k_{QDi} of the intrinsic electron relaxation constant. To account for the HT process, amplitudes B_j and number of acceptors m were allowed to vary, while the rate constant k_{HTj} was held constant, as it denotes the rate for a 1:1 cluster to QD system. The intrinsic QD dynamics and the HT process are treated independently, and thus $\sum_i A_i = 1$ and $\sum_j B_j = 1$.

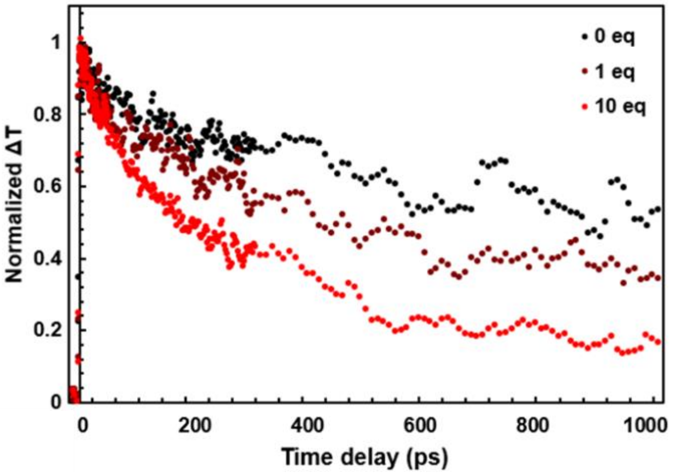

Figure S3. Normalized change in transmission (ΔT) of the $1S_e$–$1S_{h3/2}$ transition of 525 nm CdSe QDs without $V_6O_6^{-1}$ present (black) and with increasing concentration of $V_6O_6^{-1}$ as a function of probe delay.

Table S2. Fitting Parameters for TCSPC Lifetime Measurements of CdSe-TDPA QDs with $V_6O_6^{-1}$ Clusters.

**Intrinsic Relaxation Kinetics**				**Hole Transfer Kinetics**
τ_{QD1} (A_1)	τ_{QD2} (A_2)	τ_{QD3} (A_3)	$<\tau_{QD}>^a$	τ_{1HT} (B_1)
0.62 ± 0.06 ns (46.1 ± 0.4 %)	5.89 ± 0.47 ns (26.3 ± 3.2 %)	42.50 ± 3.31 ns (27.6 ± 3.5 %)	13.58 ± 2.38 ns	1.39 ± 0.09 ns (100 %)

**Hole Transfer Kinetics with Various $V_6O_6^{-1}$ Equivalents**				
**# Equiv. $V_6O_6^{-1}$**	**# acceptors**	τ_{nHT} [b]	$E_{HT}^{(lifetimes)}$	$E_{HT}^{(quenching)}$
**0.5**	0.83 ± 0.00	1.67 ± 0.10 ns	55.8 ± 0.4 %	73.9 ± 2.2 %
**1**	1.06 ± 0.31	1.31 ± 0.33 ns	69.8 ± 9.5 %	90.0 ± 6.1 %
**2**	1.58 ± 0.17	0.88 ± 0.13 ns	80.2 ± 3.2 %	93.6 ± 5.8 %
**5**	1.89 ± 0.03	0.74 ± 0.04 ns	84.0 ± 1.7 %	96.3 ± 1.0 %
**10**	2.27 ± 0.08	0.61 ± 0.02 ns	87.8 ± 0.1 %	97.7 ± 0.4 %

[a] Amplitude-weighted average lifetime $<\tau_{QD}> = \Sigma_i A_i \tau_{QDi}/(\Sigma_i A_i)$. [b] Hole transfer lifetime $(1/k_{nj}) = n(1/k_{1HT})$. All errors are estimated by the standard deviation of multiple measurements.

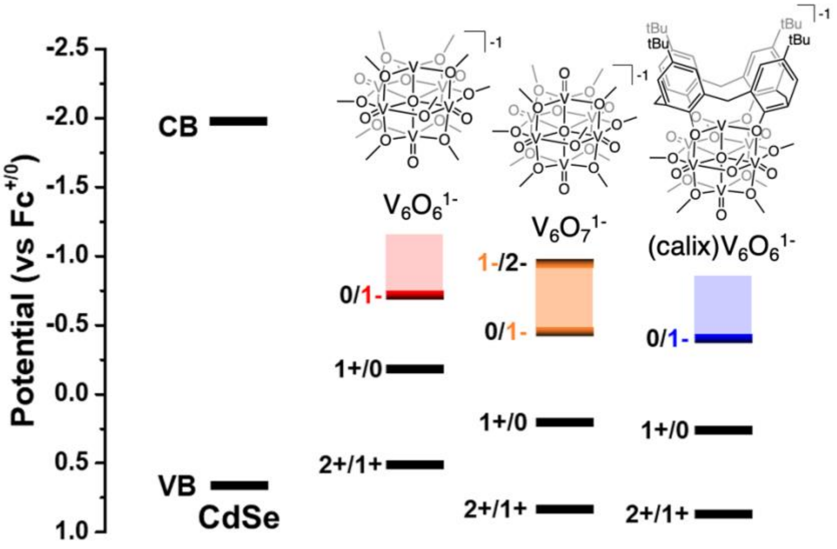

Figure S4. Conduction and valence band positions estimated for D = 2.8 nm CdSe QDs compared to the reported redox potentials for V_6O_6, V_6O_7, and (calix)V_6O_6 clusters in DCM. All potentials derived from literature or experimentally measured values (see Table S1).

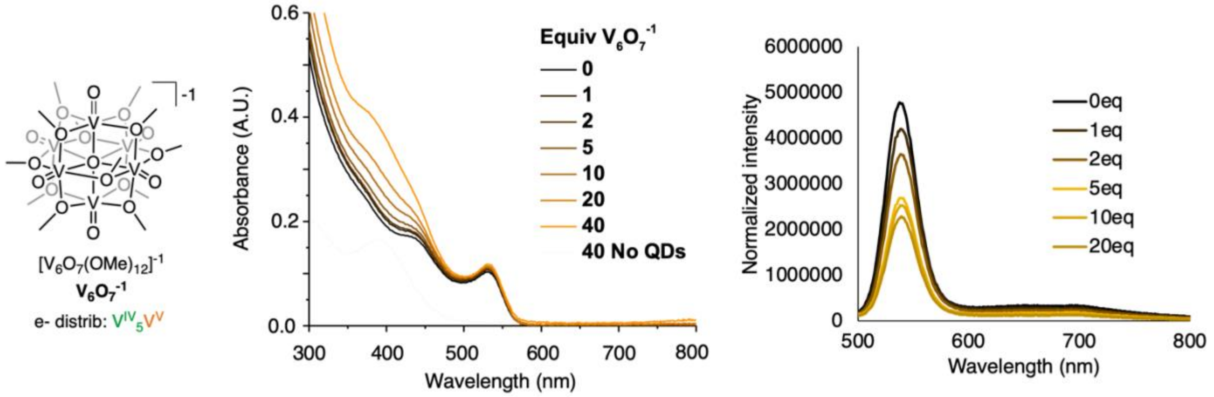

Figure S5. The absorbance and photoluminescence spectra of CdSe-TDPA with increasing concentration of $\mathbf{V_6O_7^{-1}}$ (solid black to orange); absorbance data also includes spectrum of 50 μM $\mathbf{V_6O_7^{-1}}$ in the absence QDs (dashed orange). All QD samples contained 1 μM CdSe-TDPA QDs with 0-20 equivalents of $\mathbf{V_6O_7^{-1}}$ in DCM under inert gas at room temperature.

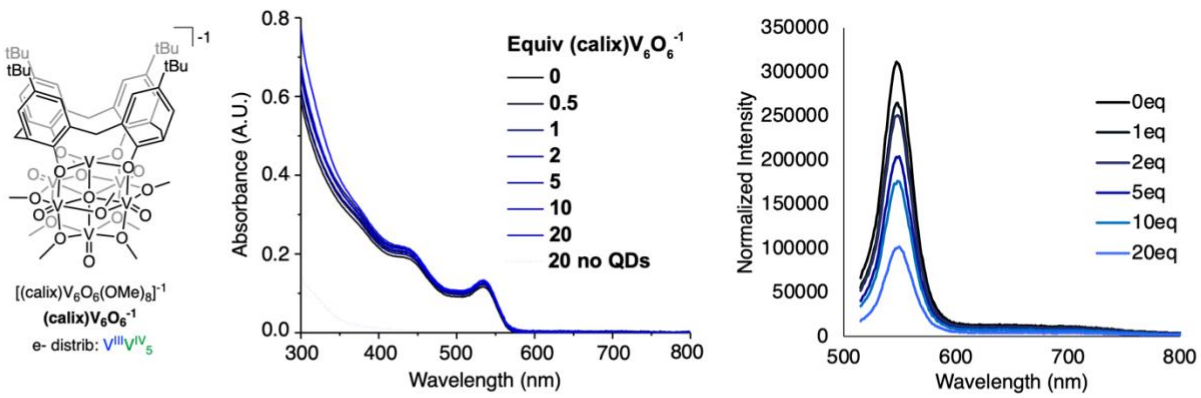

Figure S6. The absorbance and photoluminescence spectra of CdSe-TDPA with increasing concentration of **(calix)$V_6O_6^{-1}$** (solid black to blue); absorbance data also includes spectrum of as 20 μM **(calix)$V_6O_6^{-1}$** without QDs (dashed blue). All QD samples contained 1 μM CdSe-TDPA QDs with 0-20 equivalents of **(calix)$V_6O_6^{-1}$** in DCM under inert gas at room temperatue.

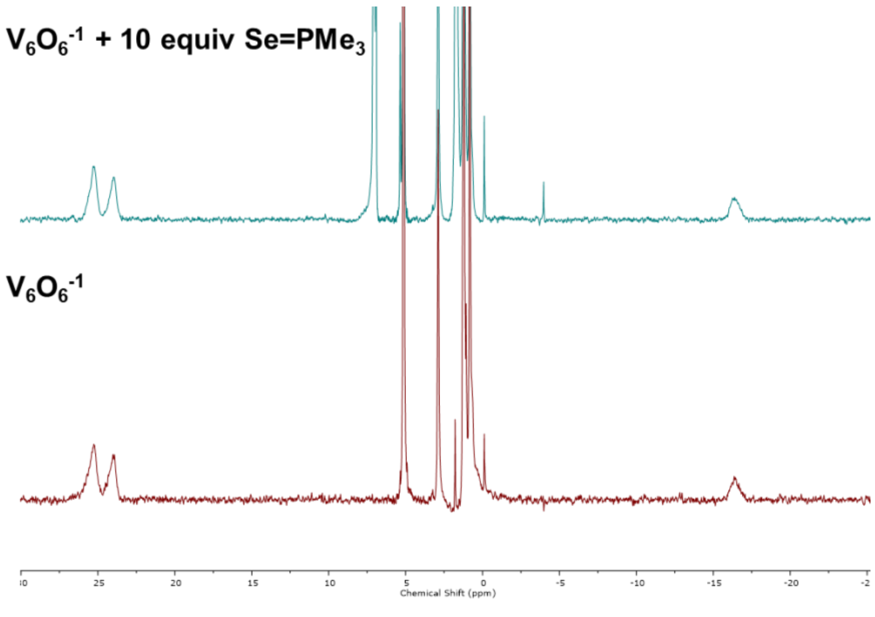

Figure S7. ^{1}H NMR Spectra for **$V_6O_6^{1-}$** with (top) and without (bottom) 10 equivalents of trimethyl phosphine selenide. All samples were allowed to sit overnight and collected in CD_2Cl_2 under inert gas. Trimethyl phosphine selenide was synthesized following established procedures.[10]

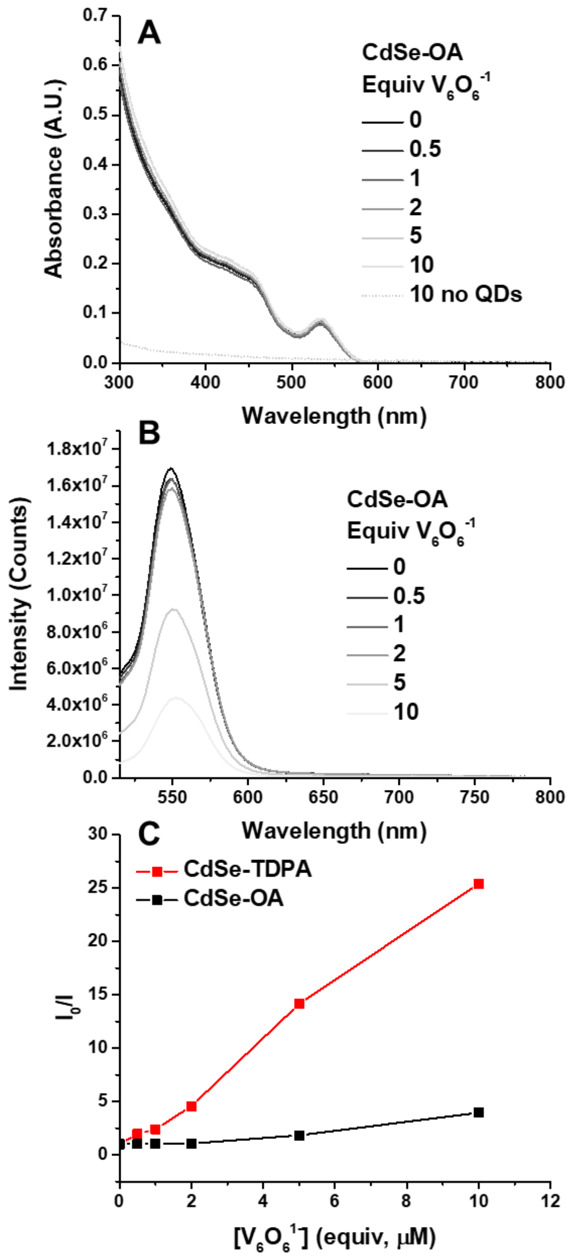

Figure S8. The absorbance (A) and fluorescence (B) spectra of CdSe-OA with increasing concentration of $\mathbf{V_6O_6^{-1}}$ (solid black to gray) as well as 10 μM $\mathbf{V_6O_6^{-1}}$ without QDs (dashed grey). (C) Comparison of the Stern-Volmer plots for TDPA- and OA-capped CdSe QDs (see **Figure 1**). All QD samples contained 1 μM CdSe-OA QDs with 0-10 equivalents of $\mathbf{V_6O_6^{-1}}$ in DCM under inert gas.

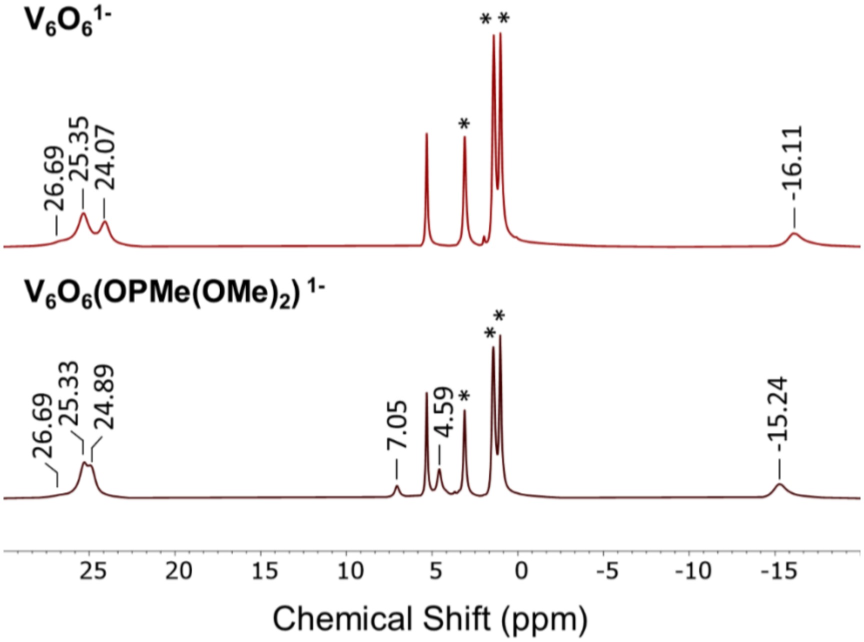

Figure S9. ^{1}H NMR Spectra for **$V_6O_6^{1-}$** (top) and **$V_6O_6(OPMe(OMe)_2)^{1-}$** (bottom). All samples were collected in CD_2Cl_2.

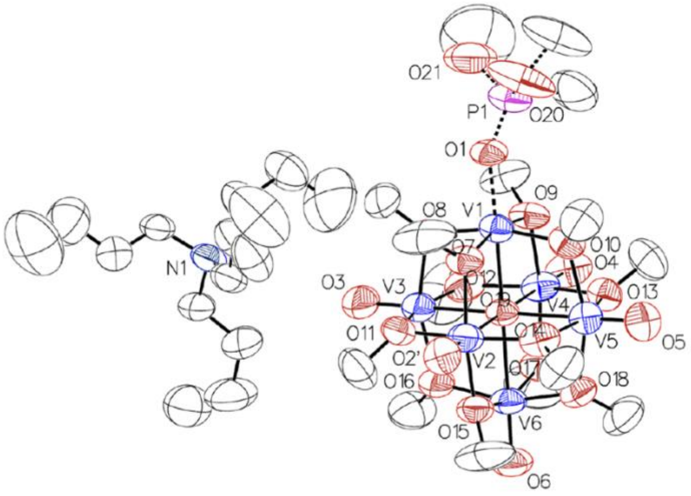

Figure S10. Molecular structure of $\mathbf{V_6O_6(OPMe(OMe)_2)^{1-}}$ shown with 30% probability ellipsoids. Solvent molecules and hydrogen atoms have been removed for clarity.

Table S3: Crystallographic parameters for the molecular structure of complexes $\mathbf{V_6O_6(OPMe(OMe)_2)^{1-}}$.

Compound	$\mathbf{V_6O_6(OPMe(OMe)_2)^{1-}}$
Empirical formula	$C_{31}H_{81}NO_{21}PV_6$
Formula weight	1140.57
Temperature / K	100.00(10)
Wavelength / Å	1.54184
Crystal group	Orthorhombic
Space group	*Pbca*
Unit cell dimensions	*a* =18.5555(3) Å<br>*b* =18.4383(3) Å<br>*c* =28.8001(5) Å<br>*α* = 90°<br>*β* = 90°<br>*γ* = 90°
Volume / Å^3	9853.4(3)
Z	8
Reflections collected	44419
Independent reflections	10841
Completeness (theta)	99.7%
Goodness-of-fit on F^2	1.032
Final *R* indices [*I*>2sigma(*I*)]	*R*1 = 0.0844
Largest diff. peak and hole	0.761 and -0.375 e.Å^{-3}

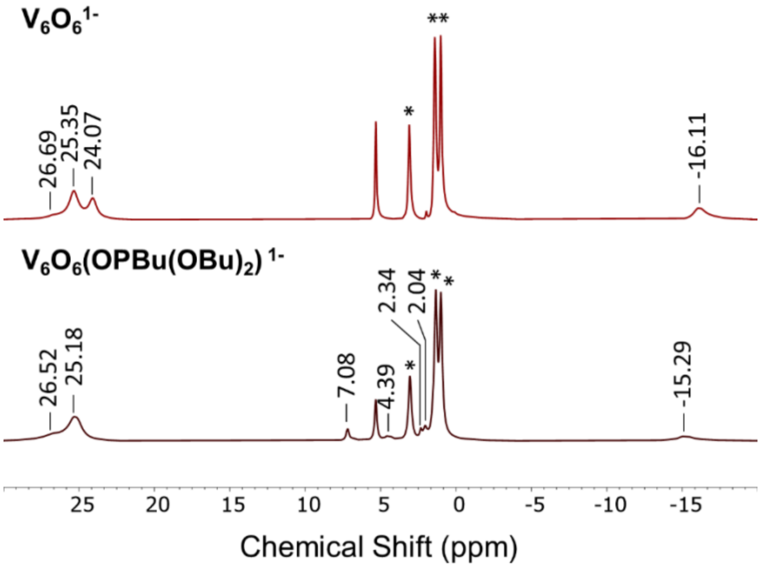

Figure S10. ^{1}H NMR Spectra for $\mathbf{V_6O_6^{1-}}$ (top) and $\mathbf{V_6O_6(OPBu(OBu)_2)^{1-}}$ (bottom). All samples were collected in CD_2Cl_2.

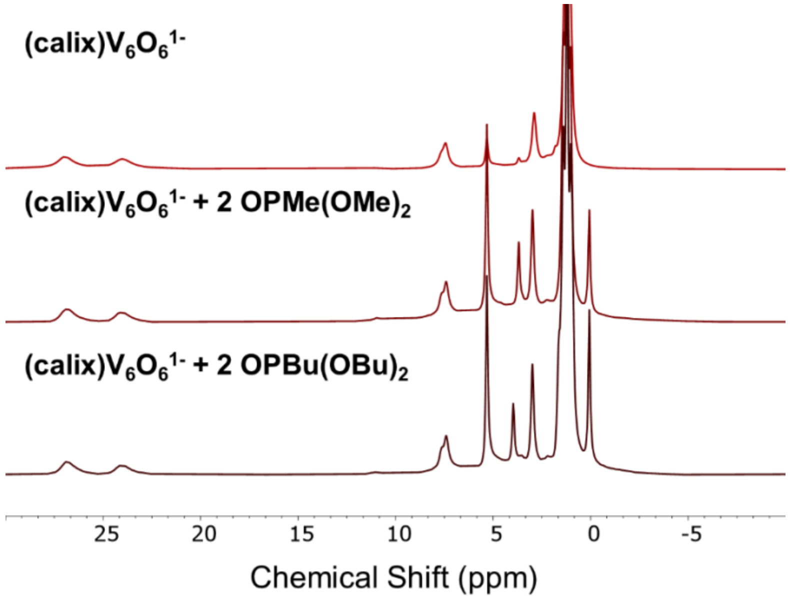

Figure S11. ^{1}H NMR spectra of **(calix)**$\mathbf{V_6O_6^{1-}}$ (top) compared to the crude spectra of **(calix)**$\mathbf{V_6O_6^{1-}}$ with two equivalents of OPMe(OMe)$_2$ (middle), and **(calix)**$\mathbf{V_6O_6^{1-}}$ with two equivalents of OPBu(OBu)$_2$ (bottom). Collecting the two crude spectra was performed by adding 2 equivalents of the respective phosphonate to CD_2Cl_2 in a J-Young tube with 1 equivalent of the cluster. The reaction was allowed to sit at room temperature for 1 hour before the ^{1}H NMR spectrum was collected. No change in the spectra is observed upon introduction of phosphonate ligand to **(calix)**$\mathbf{V_6O_6^{1-}}$, suggesting the steric bulk of the calix ligand has prevented binding to the vacant site.

References:

(1) Spandl, J.; Daniel, C.; Brüdgam, I.; Hartl, H. Synthesis and Structural Characterization of Redox-Active Dodecamethoxoheptaoxohexavanadium Clusters. *Angew. Chem. Int. Ed.* **2003**, *42* (10), 1163-1166, https://doi.org/10.1002/anie.200390306. DOI: https://doi.org/10.1002/anie.200390306 (acccessed 2021/11/19).
(2) Petel, B. E.; Brennessel, W. W.; Matson, E. M. Oxygen-Atom Vacancy Formation at Polyoxovanadate Clusters: Homogeneous Models for Reducible Metal Oxides. *J. Am. Chem. Soc.* **2018**, *140* (27), 8424-8428. DOI: 10.1021/jacs.8b05298.
(3) Yu, W. W.; Qu, L.; Guo, W.; Peng, X. Experimental Determination of the Extinction Coefficient of CdTe, CdSe, and CdS Nanocrystals. *Chem. Mater.* **2003**, *15* (14), 2854-2860. DOI: 10.1021/cm034081k.
(4) Connelly, N. G.; Geiger, W. E. Chemical Redox Agents for Organometallic Chemistry. *Chem. Rev.* **1996**, *96* (2), 877-910. DOI: 10.1021/cr940053x.
(5) Aranzaes, J. R.; Daniel, M.-C.; Astruc, D. Metallocenes as references for the determination of redox potentials by cyclic voltammetry —Permethylated iron and cobalt sandwich complexes, inhibition by polyamine dendrimers, and the role of hydroxy-containing ferrocenes. *Can. J. Chem.* **2006**, *84* (2), 288-299. DOI: 10.1139/v05-262 (acccessed 2021/12/03).
(6) Talapin, D. V.; Mekis, I.; Götzinger, S.; Kornowski, A.; Benson, O.; Weller, H. CdSe/CdS/ZnS and CdSe/ZnSe/ZnS Core−Shell−Shell Nanocrystals. *The Journal of Physical Chemistry B* **2004**, *108* (49), 18826-18831. DOI: 10.1021/jp046481g.
(7) Kosswattaarachchi, A. M.; VanGelder, L. E.; Nachtigall, O.; Hazelnis, J. P.; Brennessel, W. W.; Matson, E. M.; Cook, T. R. Transport and Electron Transfer Kinetics of Polyoxovanadate-Alkoxide Clusters. *J. Electrochem. Soc.* **2019**, *166* (4), A464-A472. DOI: 10.1149/2.1351902jes.
(8) Burke, R.; Cogan, N. M. B.; Oi, A.; Krauss, T. D. Recovery of Active and Efficient Photocatalytic H2 Production for CdSe Quantum Dots. *J. Phys. Chem. C* **2018**, *122* (25), 14099-14106. DOI: 10.1021/acs.jpcc.8b01237.
(9) Boulesbaa, A.; Huang, Z.; Wu, D.; Lian, T. Competition between Energy and Electron Transfer from CdSe QDs to Adsorbed Rhodamine B. *J. Phys. Chem. C* **2010**, *114* (2), 962-969. DOI: 10.1021/jp909972b. Huang, J.; Huang, Z.; Jin, S.; Lian, T. Exciton Dissociation in CdSe Quantum Dots by Hole Transfer to Phenothiazine. *J. Phys. Chem. C* **2008**, *112* (49), 19734-19738. DOI: 10.1021/jp808291u.
(10) García-Rodríguez, R.; Liu, H. Mechanistic Study of the Synthesis of CdSe Nanocrystals: Release of Selenium. *J. Am. Chem. Soc.* **2012**, *134* (3), 1400-1403. DOI: 10.1021/ja209246z.
